# Evaluating the Implementation of a Mental Health App for Overseas Filipino Workers in Macao China: A Mixed-Methods Study of Stakeholders’ Perspectives

**DOI:** 10.3389/fpsyt.2022.836156

**Published:** 2022-05-03

**Authors:** Andrian Liem, Karmia A. Pakingan, Melissa R. Garabiles, Hao Fong Sit, Sebastian Burchert, Agnes I. F. Lam, Brian J. Hall

**Affiliations:** ^1^Jeffrey Cheah School of Medicine and Health Sciences, Monash University Malaysia, Bandar Sunway, Malaysia; ^2^Department of Psychology, De La Salle University, Manila, Philippines; ^3^Scalabrini Migration Center, Quezon City, Philippines; ^4^Department of Psychology, The University of Hong Kong, Hong Kong, Hong Kong SAR, China; ^5^Division of Clinical Psychological Intervention, Department of Education and Psychology, Freie Universität Berlin, Berlin, Germany; ^6^Department of Communication, University of Macau, Macau, Macau SAR, China; ^7^Centre for Macau Studies, University of Macau, Macau, Macau SAR, China; ^8^Center for Global Health Equity, NYU Shanghai, Shanghai, China; ^9^Johns Hopkins Bloomberg School of Public Health, Baltimore, MD, United States

**Keywords:** digital health, telemedicine, telehealth, stepped care, global mental health, implementation science

## Abstract

**Background:**

Overseas Filipino workers (OFWs) is one of the largest communities of international migrant workers. They face systemic barriers to fulfilling their health needs. The COVID-19 pandemic worsened this condition and provided a context to evaluate the utility of a digital mental health intervention delivered within a stepped-care model to address OFW mental health. Using an implementation science framework, this study aimed to evaluate stakeholders’ perspectives on the implementation of Kumusta Kabayan, a mobile phone-based mental health app, for OFWs in Macao.

**Methods:**

A mixed-methods convergent design was used by conducting two parallel steps, including quantitative and qualitative measures. The quantitative data was collected from Filipino team members and local non-governmental organization (NGO) staff members (*N* = 12). The qualitative data were gathered from interviews with OFWs in Macao who used the app (*N* = 25; 80% females, 76% domestic workers).

**Results:**

From the online survey, the staff members of the local partner NGO and the Filipino team members strongly perceived that their organization could adapt Kumusta Kabayan to their program and generally evaluated that Kumusta Kabayan achieved its goal and was received well by OFWs. In the interviews, the OFW app users shared their experiences in using Kumusta Kabayan, which was thematically organized into six aspects of the participants’ experience: (1) promotional channel and expectation; (2) when to use the app and in what language; (3) lessons learnt; (4) memorable aspects; (5) key facilitators and barriers; and (6) suggestions.

**Conclusion:**

Kumusta Kabayan was well accepted and shows potential to be integrated into the existing support services for OFWs in Macao. This app has the promise of being scaled-up for OFWs in other countries by collaborating with local and overseas stakeholders. Lessons learnt from this evaluation could also be implemented in wider digital mental health services in different settings.

## Introduction

Overseas Filipino workers (OFWs) is one of the largest international migrant worker communities, with an estimated number of over 10 million worldwide (1). They work in both high-skill and low-skill sectors across countries to improve their lives and the livelihoods of their family members who remain in the Philippines (2). However, OFWs and other migrant workers struggle to fulfill their health needs because of systemic barriers they experience including language difficulties, adverse working conditions, lack of available healthcare providers, and inadequate social protection for migrant workers in the host countries (3, 4). Consequently, migrant workers, including OFWs, experience higher risks for developing mental disorders like anxiety and depression than local people in the host countries (5–7).

The COVID-19 pandemic worsened this condition as migrant workers were forced to take temporary unpaid leaves from employment or suddenly lost their jobs and social protections (8, 9). Additionally, OFWs and other migrants who worked as domestic workers encountered other challenges like excessive work load from their employers who began to work from home and some were not permitted to take their normal day-off due to employer’s fears of COVID-19 contamination and lack of trust in the domestic workers’ hygiene (10, 11). Due to cultural and language barriers, migrant workers were also at risk of having inadequate knowledge and awareness of the latest COVID-19 situation and policies in their host countries (12). Consequently, OFWs and other migrant workers’ mental health deteriorated and they have to survive with the limited mental health support in the host countries (13).

The inequity in healthcare access during the pandemic may be reduced by implementing digital mental health interventions within a stepped-care approach, which includes providing the least intensive intervention based on clients’ needs and monitoring them regularly (14). Digital mental health interventions, including *via* the internet and mobile phones, were found to be consistently effective in reducing mental disorders like depression and anxiety (15, 16). Furthermore, mobile phone-based health apps can also accurately screen mood disorder symptoms (17). Furthermore, a previous study on OFWs’ attitude toward digital health interventions found that the community showed their acceptance and willingness in accessing mobile phone-based mental health interventions (18). However, mobile phone-based mental health applications for improving mental health of OFWs and other migrant workers were still limited as previous interventions were mostly developed for inpatient adults (15).

With the limited internet and mobile phone-based mental health interventions for non-clinical groups, the World Health Organization (WHO) developed Step-by-Step, an online guided self-help intervention for people with depressive symptoms (19). Step-by-Step is an online version of the WHO’s offline transdiagnostic psychological intervention for common mental health problems named Problem Management Plus (PM+) (19, 20). The Step-by-Step website version has been piloted among Lebanese, Palestinian, and Syrian adults in Lebanon (21) and the mobile application version was tested among adult Syrian refugees residing in Germany, Sweden and Egypt (22). These studies showed that Step-by-Step was a promising stepped-care digital mental health intervention for OFWs. The Step-by-Step program was also culturally adapted for use among Filipino overseas workers.

**FIGURE 1:**
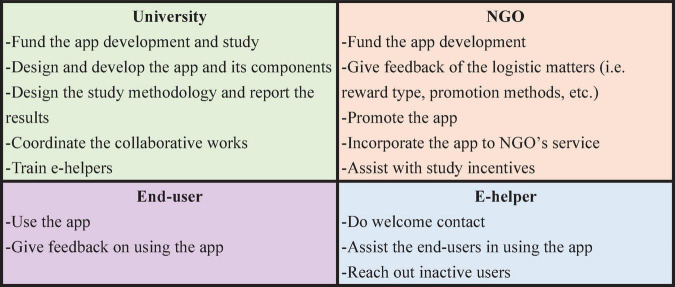
Stakeholder members’ roles.

The current study evaluated stakeholders’ perspectives on the implementation of Step-by-Step for OFWs in Macao using a mixed-methods design. The study utilized an implementation science approach, which emphasized the practical aspects of how to scale-up an evidence-based intervention (24). The current study presents the results of the implementation evaluation of the program utilizing questionnaire data collected from our local non-governmental organization (NGO) partner and e-helpers (digital lay health workers) in the Philippines as well as interviews with OFWs in Macao as end users of the Filipino version of Step-by-Step named Kumusta Kabayan, which means “Hello, fellow Filipino.” The roles of each stakeholder member are presented in Figure 1 below. Using an implementation science framework, this study was designed to accelerate the translation of evidence-based intervention into real-world system of care and address inequities in healthcare delivery for migrant workers (25, 26).

## Materials and Methods

This implementation science evaluation study used a mixed-methods parallel convergent design (27). The evaluation was conducted in two parallel steps, using quantitative and qualitative measures, which complemented each other and provided more comprehensive evaluation results (28). Details of the study is described in sub-sections below. Ethical approval for the study was granted from the Research Ethics Committee at the University of Macau (SSHRE19-APP074-FSS).

### Sampling and Procedure

For the quantitative measure, total population sampling (29) was used where the lead author sent message invitations to the anonymous online evaluation survey to all e-helpers, clinical supervisors, staff and volunteers of the local NGO partner. The online survey for staff and volunteers of the local NGO partner were provided in English and Chinese to increase the participants’ understanding. Before completing the survey, electronic informed consent forms were provided to the participants.

**FIGURE 2:**
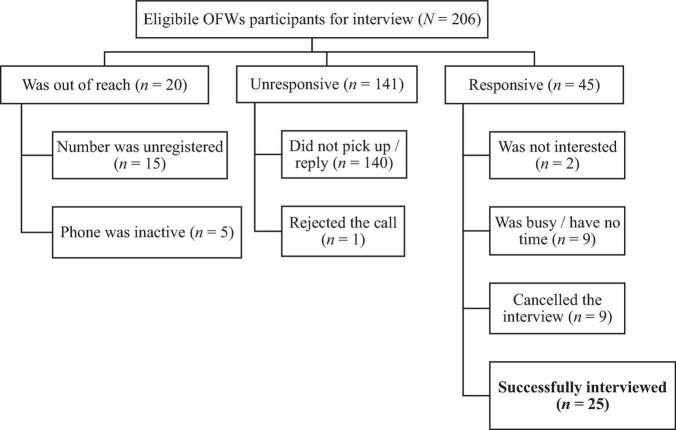
Participants recruitment for interviews from both completer and non-completer groups.

For the qualitative measure, participants were recruited with a convenience census-approach sampling. All participants who participated in the Kumusta Kabayan (*N* = 206) and were screened with the Patient Health Questionnaire (PHQ)-9 for depressive symptoms (6) were considered as eligible respondents and were asked if they were willing to participate in the interview. Participants who either completed or did not complete the program were purposively invited to represent diverse experiences. Not all potential interviewees joined the interviews as illustrated in Figure 2.

Before the interviews were audio-recorded, participants were informed about the aim of interview and verbally asked for their consent. One-to-one audio interviews from October 2020 to February 2021 were conducted by e-helpers using voice over IP services. Interviews lasted an average of 25 min (SD = 8) and were conducted in English or Tagalog. Participants were compensated with a food voucher worth MOP 100 (≈USD $12) for their time. In order to improve participants’ openness, they were assigned to a different e-helper for the interview if they were previously assisted by an e-helper during the intervention period. Moreover, e-helpers were also trained for doing the interviews using a role play method prior to the data collection.

### Participants

The demographic information (e.g., age and sex) of participants in the survey (*N* = 12) was not asked to protect their anonymity, given the small sample sizes of e-helpers and NGO partner members. Participants in this quantitative measure consisted of a team in the Philippines (*n* = 8), including two clinical psychologists, two Master of Psychology students, and four Bachelor of Psychology students; and a team in Macao (*n* = 4), including three social workers and one logistical staff.

The interviewed OFW participants’ demographic information (*N* = 25) was retrieved from their biodata in the app. The interviewed participants were mostly female (*n* = 20), with an average age of 39 years old (SD = 8), and dominated by a domestic work job (*n* = 19). On average, they have been working as OFWs for 59 months (SD = 78), having 66 working hours per week (SD = 16), and with a monthly salary of MOP 4,664 (SD = 1,182) (≈USD $580, exchange rate in July 2021). The interviewed participants consisted of both completers and non-completers. The demographic information of interviewees’ and non-interviewees were comparable (Supplementary Material).

### Instruments

The online survey was adapted from previous studies on evaluation of innovative health programs promotion and implementation (30–33). The measures included organizational climate scale, awareness and concern scale, perceived advantage scale, perceived complexity scale, and level of success scale. Also, one open-ended item to ask for general feedback and suggestion was located at the end of the online survey (see Supplementary Material).

The 11-item organizational climate scale consisted of three parts: mission, teamwork, and infrastructure support. An item from this measure is “Our team is willing to take a chance on a good idea” that was measured with Likert’s scale from 1 (strongly disagree) to 5 (strongly agree), where higher summed scores represent more positive organizational climate in adapting a new program. This scale was given only to the team in Macao due to the nature of the items. The internal reliability (Cronbach’s alpha) of this measure was 0.94. The 9-item awareness and concern scale consisted of three parts: awareness, concern, and interest. An item example of this measure is “I am aware that Kumusta Kabayan addresses depressive symptoms.” Item responses were on a Likert-type scale from 1 (not at all true) to 4 (very true), where higher summed scores represent higher awareness and concern of participants to the program. The Cronbach’s alpha of this measure was 0.77.

The perceived advantage scale was measured with a 3-item Likert scale from 1 (strongly disagree) to 5 (strongly agree), where higher summed scores represent greater perceived level of program’s advantage. An item from this measure is “The Kumusta Kabayan program can improve the mental health of Filipino workers in Macao.” The Cronbach’s alpha of this measure was 0.71. The perceived complexity scale was measured with a single item of organizational level (Part A, “The Kumusta Kabayan would be difficult for our organization/institution to explain to potential Filipino migrant worker users”) and 2-item Likert scale (Part B) from 1 (strongly disagree) to 5 (strongly agree), where higher total score represents higher perceived complexity of the program. A sample item from Part B is “The Kumusta Kabayan program would be hard for Filipino migrant workers to understand.” The Cronbach’s alpha of this measure (Part B) was 0.74. These reliability coefficients around 0.70 are considered moderately reliable (34). The last question, level of success, asked the participants to choose one of eight continuum options based on their experience and observation. It started from Option 1 (The Kumusta Kabayan program not only failed to meet its goals, but it caused a loss of resources and create other problems) to complete successful perception of Option 8 (The Kumusta Kabayan program fulfilled all of its goals and also provided other benefits for Filipino migrant workers and/or for other stakeholders).

For the qualitative measure, all e-helpers followed the interview guide (Supplementary Material) that was developed for this study. The guideline was pilot tested among e-helpers and with three participants. During the interviews, e-helpers introduced themselves and asked for participants’ verbal consent. Consented participants were asked about the following themes: referral channel and initial thoughts about the program, period of use, lessons learnt, key facilitators and barriers, and improvement suggestions for the program.

### Data Analysis

Results from the online survey were descriptively analyzed. The inferential analysis was not conducted due to the limited number of responses and demographic variables of online survey participants were not collected to protect participants’ anonymity. Data from the research team in Macao and the Philippines were visually compared to show any contrast or similarity between the stakeholders in evaluating the program.

Interview audio-recordings were transcribed by the e-helper who did the interview within 2 weeks from the interview then sent to the clinical supervisor and research coordinators. Following a Rapid Evaluation and Assessment Method (35) that was used in a similar evaluation study (36), the lead author with a doctoral degree in psychology and mixed-method research experience read each transcript and summarized the information into a table for each participant using a deductive content analysis approach (i.e., promotional channel and initial expectation). This summary table was sent to other research team members and clinical supervisors for iterative analysis and discussion using an inductive content analysis method, in which the interpretation focused on the explicit data given by the participants (37, 38). Steps in this method included open coding, creating categories, and abstraction for each aspect. Data saturation was reached in the interview with the 14th participant where the responses shared similarity with previous responses. The Consolidated Criteria for Reporting Qualitative Studies (COREQ) (39) was used as a guideline for the qualitative portion of the study.

### Reflexivity

This study was part of an ongoing implementation study that involved research team members from multiple sectors and countries that might have different perspectives and cultural backgrounds. Therefore, at the beginning of the study, regular intensive meetings were conducted to build mutual understanding and improve transparency, including the challenges that might be faced and alternative solutions. Along the way, regular meetings were also conducted among e-helpers, clinical supervisors, and research coordinators to discuss any experiences and reflective thoughts during the interviews. The research coordinators emphasized that all opinions shared within the meeting were valid and important to encourage members to share both positive and negative experiences. The e-helpers also had their regular meetings with their clinical supervisor, in which the information in the minutes could be anonymized when necessary so they could openly share their thoughts without worry of being judged. The same step was also conducted when collecting quantitative feedback by using an anonymous online survey.

## Results

Findings were presented in the two sub-sections below. The quantitative results from local NGO staff members and research team members in the Philippines (*N* = 12) cover organizational climate, awareness and concern, the app’s advantage and complexity, and level of success. Findings from the interviews with OFWs in Macao (*N* = 25) cover six aspects from promotional channel and expectation to suggestions.

### Online Evaluation of Local and Overseas Partner Member Perspectives

Table 1 displays the quantitative findings from the local NGO partner and overseas research team members. The median score of organizational climates from the teams in Macao and the Philippines are 4 and 5, respectively. The local partner NGO staff members and overseas members strongly perceived that their organization could positively incorporate Kumusta Kabayan into its program. However, staff members in Macao indicated their worry on infrastructure support, with a relatively lower score on this sub-scale than on other sub-scales and when compared with e-helpers in the Philippines. On the awareness and concern, both groups were highly aware of the app’s purpose, with score median of 4 in Macao and the Philippines. Both groups agreed that Kumusta Kabayan would be helpful for the OFWs. However, their perception toward the complexities of the app was ambiguous. For level of success, the teams in Macao and the Philippines generally evaluated that Kumusta Kabayan had achieved its goal.

**TABLE 1:** Characteristics of participants in online survey.

Scales & parts	Macao (*n* = 4)			The Philippines (*n* = 8)			Total (*N* = 12)		
			
	*M*	SD	Med	*M*	SD	Med	*M*	SD	Med
Organizational Climate^A^	4.00	0.00	4	4.63	1.06	5	4.42	0.90	5
Mission	4.83	0.19	5	n.a.	n.a.	n.a.	n.a.	n.a.	n.a.
Teamwork	4.15	0.25	4	4.45	1.25	5	4.35	1.01	5
Infrastructure support	3.67	0.61	4	4.17	1.10	5	4.00	0.96	4
Awareness and Concern^B^	3.53	0.53	4	3.92	0.15	4	3.79	0.36	4
Awareness	3.58	0.50	4	3.92	0.15	4	3.81	0.33	4
Concern	3.38	0.95	4	3.97	0.09	4	3.77	0.58	4
Interest	3.75	0.50	4	3.81	0.53	4	3.79	0.50	4
Perceived Advantage^A^	3.75	0.32	4	3.88	0.71	4	3.83	0.59	4
Perceived Complexity (a) – Organizational level^A^	3.00	1.40	3	2.40	1.40	2	2.58	1.38	2
Perceived Complexity (b) – Individual level^A^	2.65	1.00	3	1.75	0.65	2	2.04	0.88	2
Level of Success (hierarchal)	*f*	%		*f*	%		*f*	%	
1. The Kumusta Kabayan program not only failed to meet its goals, but it caused a loss of resources and create other problems.	0	0		0	0		0	0	
2. The Kumusta Kabayan program achieved none of the goals.	0	0		1	12.5		1	8.3	
3. The Kumusta Kabayan program achieved one or two goals.	1	25.0		0	0		1	8.3	
4. The Kumusta Kabayan program achieved none of its goals, but some Filipino migrant workers wish to continue its use.	1	25.0		0	0		1	8.3	
5. Although the Kumusta Kabayan program achieved none of its goals, but it provided other benefits for Filipino migrant workers.	0	0		0	0		0	0	
6. The Kumusta Kabayan program achieved one or two goals but caused other problems.	0	0		4	50.0		4	33.3	
7. The Kumusta Kabayan program achieved all of its goals.	1	25.0		1	12.5		2	16.7	
8. The Kumusta Kabayan program fulfilled all of its goals and also provided other benefits for Filipino migrant workers and/or for other stakeholders.	1	25.0		2	25.0		3	25.0	
	*M*	SD	*Med*	*M*	SD	*Med*	*M*	SD	*Med*
Average	5.5	2.4	5	6.1	1.9	6	5.9	2.0	6

*A = From 1 (strongly disagree) to 5 (strongly agree); B = From 1 (not at all true) to 4 (very true).*

### Interviews With Overseas Filipino Workers in Macao

Findings from the interviews with the completers and non-completers are organized into six aspects of the participants’ experience, including (1) promotional channel and expectation; (2) when to use and in what language; (3) lessons learnt; (4) memorable aspects; (5) key facilitators and barriers; and (6) suggestions. Relatable quote(s), regardless of participants’ completion status, were provided for each aspect with information of participant’s number at the end of the quote.

#### Promotional Channel and Expectation

Participants learned about the program from several promotional channels including social media like Facebook and flyers that were distributed in churches and restaurants where OFWs gather on weekends (Supplementary Material). Some of them read the news about about Kumusta Kabayan’s launching and talked about it with friends. Participants were also informed about the program from The Philippines Consulate in Macau and the local NGO partner, Caritas Macau. Participants commonly expected that the program could assist them in managing emotions, sharing experiences, or connecting with other OFWs, particularly because they recognized the name of the program in Tagalog. However, some participants who learned about the program from the Consulate thought that Kumusta Kabayan was part of the employment and visa process or it would provide monetary aid.

*“I just knew about this application from my friends here in Macao so I installed it like them. My first impression about the Kumusta Kabayan is that it would be helpful for us, our fellow Filipinos to check up on each other, to check up on how they are doing. At first, I really do not know. But, while reading the questionnaires, it is really related to the lives of the OFW and also to our personal life, so in a way it touches us*.” (Participant #94).

#### When to Use and in Which Language

Participants who completed the program typically used the app in the morning before work, sometimes in the afternoon, or at night before sleeping, depending on their free time. They used the mobile app almost every day to check for new content or use the mood tracker. Participants who partially completed or discontinued the program used the app only on their day-off on weekends. However, during weekends, they still had difficulties finding time to use the app as they were also busy with extra works or were too tired. Both English and Filipino language versions were used by participants. Some words related to psychological questions were reported hard to understand, especially in English.

“*Sometimes I was able to open it but I did not continue answering*… *Recently I wasn’t able to use it because I got busy with my work.*… *When I answer it, when I want to finish the questionnaire, there are times that I wasn’t able to finish when my boss calls me, and also when their child [who I take care of] needs me. I answer it during my rest hour. When the child is sleeping, that’s when I open it.*” (Participant #28).

“*English, for me it’s easy. But, I can’t speak for other. Ay Ineng [young girl], I don’t want to sound overconfident. But, I somehow understand the questions since I’m also a college graduate. That means I understand it. But, for those that didn’t aim a higher degree, they may not be able to answer it well. I’m not generalizing. But, in my opinion, the question and the English are okay. It’s easier for me to understand if it’s in English.*” (Participant #40).

#### Lessons Learnt

Participants explained some lessons that they learnt from the program. The basic lesson is doing reflective thinking or self-checking and managing their emotions and feelings. Participants learnt how emotions and feelings are connected to their physical state so they also understand why doing exercise is important. When feeling stressed, participants learnt from the app to divide their focus and to do self-care by doing activities they enjoy like cooking or applying breathing exercise. Moreover, they learnt the importance of making, opening themselves to others, and finding support from the local society.

“*Before when I hadn’t installed the Kumusta Kabayan, I was always stressed. I don’t go out of the boarding house. I just go from work to home all the time. But after opening Kumusta Kabayan, it was a huge help for me especially the things I’m reading from the app, including the questions from Kumusta Kabayan. There were times, when I was reading the questions or stories, then suddenly I started crying. I said to myself “This Kumusta Kabayan is such a big help for me because before I’ve forgotten to interact with other people. But this time, I’m doing well. Now I’m able to socialize with other people all because of Kumusta Kabayan.*” (Participant #207).

#### Memorable Aspects

Participants felt grateful for the program that they never knew existed before joining the study. The name of the program, Kumusta Kabayan, also made it easy for participants to understand the program’s aim. They praised the story and the characters that highly resembled participants’ lives as OFWs. Having someone like them overcome life’s challenges as an OFW made them hopeful and boosted their confidence. The psychological questions encouraged participants to reflect and disclose their feelings to their friends, including things they never told others before. From the content, participants remember breathing exercises that they could apply in their daily lives, especially when they get stressed.

“*What I like about the app are the stories that are really, sometimes it gives me a boost of self-confidence most especially that we live alone and slowly losing hope. But after reading the stories there is still hope. Something like that, we just have to keep fighting. You can do it. It’s just a problem and it will eventually end. Just like the saying that goes in every problem there is a solution.*” (Participant #50).

“*Contents related to my experiences, experiences of an OFW*… *about missing your family, and also stress.*… *and there were times when you encountered a misunderstanding with your employer. Something like that, those kinds of situations. I liked the story, I feel excited also. The character [Ate Sonia] is very inspiring too. The activity is very relaxing, especially the exercising part, Inhale-Exhale [breathing exercise]. It helped me relax my mind.*” (Participant #194).

#### Key Facilitators and Barriers

The main facilitators for successful implementation of Kumusta Kabayan were the accessibility of this app on Play Store and App Store, and the app’s small size. On another side, four key barriers for participants using the app were recognized. First is the time barrier that the majority of participants were migrant domestic workers with only one day-off. Some of them had extra work to do during their day-off and some used it for resting, and they had minimal time to prioritize the app. Second are technological barriers including lack of phone memory to accommodate the app, incompatible operating system, poor signal reception, limited or no mobile data, limited or unstable Wi-Fi, and technical glitch where the app always return to the start and did not record their responses to questionnaires. Third is the lack of the digital literacy where participants changed their phone or formatted it so they had to re-install the app. However, they could not re-install by themselves and needed assistance by research assistants. Fourth are human factors including forgetting their username or password that was case sensitive, changing phone numbers so they could not receive access code when they forgot username or password, and psychological barriers because the questions were triggering of homesickness, as explained by Participant 94 below:

“… *while answering the questionnaire, we are also in another country, it feels difficult, you really take in the emotions of each question that you will be answering. It is like you are going back to the time before you leave the Philippines, you are thinking about the past and how can you be better compared before.*” (Participants #94).

#### Suggestions

Participants suggested several points for the improvement of Kumusta Kabayan, namely (1) connectivity, (2) connectedness, (3) content, (4) feedback or brief explanation on participants’ responses to the questionnaires, (5) add-ons, and (6) promotional methods. First was about connectivity, which they recommended to connect the app with their social media account so they do not need to remember another username and password. Participants also shared their expectation for Kumusta Kabayan that does not require an internet connection because they did not always have mobile data or Wi-Fi. However, on another side, some participants disclosed that their mobile phone memory was full so they had to use the website version. Second was about connectedness, where participants would like to see a video call feature with the e-helper and group chat feature with other participants. This suggestion was motivated by participants’ need to be connected and to share stories with other OFWs and people in their home country, which would ease their homesickness. Third was about the content, which participants suggested to also integrate their positive and uplifting and positive experiences of being OFWs besides the struggles to balance the negative feelings as illustrated below. In addition, participants who came from various occupational backgrounds recommended making several storylines and characters that users could choose base on their type of job. Fourth was about feedback or brief explanation on their responses to the questionnaires so they could understand their condition. Fifth was about add-ons, which participants suggested to add entertainment features and news on the app so they still could use the app while waiting for the next session to be opened. Sixth was about the promotional methods, which participants suggested to involve the OFWs who are also YouTubers or TikTokers to promote Kumusta Kabayan because many OFWs watch these social media celebrities.

“… *maybe give many examples of the OFWs’ lives, not only the sad stories.*… *they should put stories about the bright side of being an OFW so that people will be encouraged to use the application.*… *because sometimes when the story is sad, the delivery of it is also negative, it should be something nice too.*” (Participant #28).

## Discussion

This study evaluated the stakeholders’ perspectives on the Kumusta Kabayan implementation for OFWs in Macao using a mixed-methods design. Three stakeholders were involved in this study: staff members of local NGO partner, research team members in the Philippines, and OFWs in Macao as the app users. The quantitative measure among NGO staff members and Filipino team members found that Kumusta Kabayan was perceived fit to be integrated into their organizations. In particular, the NGO staff members strongly agreed that Kumusta Kabayan was in line with the NGO’s aim of assisting migrant workers.

On the other hand, infrastructure support was a concern to both NGO staff members and Filipino members. Infrastructure support covered the available organization’s equipment and technology, human resources, and financial situation. Their concerns might be associated with the sustainability of the program as staff numbers in the NGO were limited and Filipino e-helpers were volunteer students. Moreover, the app maintenance fee was relatively high (≈USD $2,367/year) for the NGO.

Generally, the NGO staff members and overseas members perceived that Kumusta Kabayan achieved its goals. From the survey, some Filipino members chose an evaluation option that despite this success, the app caused other problems. This finding might be associated with the difficulty experienced by users when they forgot their passwords. For safety reasons, a one-time-password (OTP) would be sent to user’s phone number registered in the system if user forgot their password. However, some app users changed mobile phone numbers during the program so they could not retrieve the OTP and could not access their account privacy settings. This unfortunately led to users being unable to use the app in some instances. Future iterations of the application should provide easier and more stable access to the application.

Moreover, the OFWs also used a variety of smartphone brands and not all of the operating systems were compatible with the app. Users with inadequate digital literacy could not follow the instructions to check their smartphone configuration nor to download and install the app directly which frustrated them. This finding reflected similar results in Step-by-Step testing among refugees in European countries where technical literacy was the main barrier for them for accessing the app (22). Therefore, OFWs in the interviews suggested an easier log-in method such as linking it with their social media accounts. However, this recommendation should be thought through carefully so it will not compromise user’s data privacy. An alternative is posting FAQs, including the steps in accessing the app, in the Kumusta Kabayan program’s social media account.

Users also suggested that video calls and group chat features should be incorporated into the app. This suggestion might be driven by social isolation and loneliness among people from communal cultures (40). Moreover, the mobility restriction during the pandemic amplified the social isolation because migrant workers, particularly the migrant domestic workers, were not allowed by their employers to take the day-off or asked to stay at home during their day-off (9). This also explains why users were very touched and appreciative of the storyline because it was relatable and educational. This finding highlighted the importance of cultural adaptation as the storyline was developed carefully by involving the OFWs themselves in the adaptation process (23). However, from the interviews it was found that some users would like to see more characters from different types of jobs because of different challenges faced by these OFWs.

In the interviews we also explored the main barriers for OFWs when using the app, including from the users who discontinued using it. The primary reason was they did not have time to use the app because they were busy with their jobs, which some also did extra work during their day-off. The demographic information showed that our participants could work up to 14 h per day on average. OFWs who worked as domestic workers had difficulty opening the app because they were also taking care of the children or older adults in the family. This finding supported previous study on migrant workers’ health that migrant domestic workers were at high risk for injuries, mental problems, and low quality of life because of long working hours, low wages, and inadequate health insurance (41). Furthermore, the working condition of OFWs shared by the users also explained their suggestion about entertainment add-ons so they could relax when using the app. Moreover, many users opened the app at night after long working hours and could not concentrate on reading on their screen. Therefore, a format of video or animation could be an alternative in delivering the content for OFWs.

Lastly, the interviews enlightened us about the promotional channels and methods. It was clear that using social media as an online method and face-to-face promotion at church and restaurants worked well in recruiting potential users. Moreover, collaboration with the local NGO that was well-known for its programs in assisting migrant workers enhanced the trust of OFWs for the app. However, when the app was launched and covered by the mass media, the local people showed divided attitude toward this program as some viewers commented that the citizens should be taken care more than the foreigners [i.e., (42)]. This resistance and sentiment toward migrant workers confirmed a previous study on fear and disgust toward migrant workers, particularly domestic workers, as their professions were perceived as low-end 3D jobs (dangerous, difficult, and dirty) (43). The insight emerged from the interviews was to collaborate with OFWs who were popular YouTubers and TikTokers to promote the app on their channels. Also, the interviews revealed that collaborations with the Government’s representatives either from the host country or sender country, was like a double-edged sword because it could reach many OFWs but not all of them trust these entities and were thinking that the app was a surveillance program.

### Implications for Practice and Future Studies

The findings from this study could be extended to the improvement of mobile mental health apps implementation, particularly for migrant workers. Moreover, lessons learnt from this evaluation could also be useful to be implemented in wider digital mental health services for other group of users. For example, the very appreciated storyline and characters were designed from a cultural adaptation process, which also being a critical step for in designing digital health intervention for Chinese young adults (44). The local partner and Filipino e-helpers contributed substantially to the successful implementation of Kumusta Kabayan. Therefore, it is also important to involve the stakeholders at the earliest possible time during the planning phase, to minimize the potential barriers in implementing digital mental health. Also, it is crucial to assess the digital literacy level of potential users when designing the app as having a smartphone does not guarantee that the owner could use it easily.

Another practical implication, the users’ recommendations of adding additional features including feedback on questionnaires, video chat, group chat, entertainment, and news could be integrated with gamification principles to improve user engagement and increase retention (45). For example, an app user might creatively design an avatar for their profile picture that would increase the connectedness to the app. Leaderboard and progress bar (46) could also be introduced to encourage users to complete the challenges during the sessions such as planning and executing the social activities as a part of behavioral activation intervention. Users might collect virtual coins from completing activities or just from opening the app that these coins can be used to access the suggested add-ons. Future studies could investigate the effectivity of these gamification principles in mobile phone app for migrant workers. Additionally, a future study could explore the virtual connections among app users within the app online community using a digital ethnography method (47).

### Limitations

This study has two limitations. First, despite its anonymity, evaluation from the local and overseas partners relied on self-report questionnaires that might lead to social desirability. Second, despite data saturation and engagement with discontinued users, evaluation from the OFWs might be affected by self-selection bias as disappointed users might ignore the interview invitation so their perspective was less represented. Regardless of these limitations, the quantitative and qualitative findings in our study were complimentary of one another and provided key directions to improve subsequent implementation of Kumusta Kabayan among OFWs in Macao and elsewhere.

## Conclusion

This study reported the implementation evaluation of Kumusta Kabayan, a mobile mental health app for OFWs, from the perspective of stakeholders including staff members of local NGO as a collaborator, team members in the Philippines, and OFWs in Macao as the app users. The Kumusta Kabayan program was accepted by the users because the character’s story highly resonated with their lives as OFWs and the app was relatively easy to use. The Kumusta Kabayan has the potential of being integrated into the existing support services for migrant workers in Macao by collaborating with the local NGO stakeholder. This app could be scaled-up for OFWs in other countries by paying attention to the key facilitators, barriers, and the suggestions shared by the users that reported in this study.

## Data Availability Statement

The raw data supporting the conclusions of this article will be made available by the authors, without undue reservation.

## Ethics Statement

The studies involving human participants were reviewed and approved by the ethical approval for the study was granted from the Research Ethics Committee at the University of Macau (SSHRE19-APP074-FSS). The patients/participants provided their written informed consent to participate in this study.

## Author Contributions

AL, HS, and BH: study conceptualization and design. AIFL and BH: secured funding. AL, AIFL, BH, HS, KP, MG, and SB: data collection and final draft writing, review, and editing. AL, KP, and MG: data analysis. AL: draft manuscript writing. All authors contributed to the article and approved the submitted version.

## Conflict of Interest

BH is co-editing the Frontiers special topic “Global mental health among marginalized communities in pandemic emergencies” but was not directly involved in the review of this article. The remaining authors declare that the research was conducted in the absence of any commercial or financial relationships that could be construed as a potential conflict of interest.

## Publisher’s Note

All claims expressed in this article are solely those of the authors and do not necessarily represent those of their affiliated organizations, or those of the publisher, the editors and the reviewers. Any product that may be evaluated in this article, or claim that may be made by its manufacturer, is not guaranteed or endorsed by the publisher.
