# Supplementary material for: Evaluating the Implementation of a Mental Health App for Overseas Filipino Workers in Macao China: A Mixed-Methods Study of Stakeholders’ Perspectives
Source: Front Psychiatry. 2022 May 3;13:836156. doi: 10.3389/fpsyt.2022.836156 (PMC9113052; doi:10.3389/fpsyt.2022.836156)
Supplement: Supplementary file 1 [file Data_Sheet_1.pdf]

FULL TITLE

Evaluating the implementation of a mental health app for overseas Filipino workers in Macao China: A mixed-methods study of stakeholders' perspectives

SHORT-TITLE

Mental health app implementation evaluation

TYPE & VERSION

Original research article, Version 1

AUTHORS & AFFILIATIONS

1. Andrian Liem
   Jeffrey Cheah School of Medicine and Health Sciences, Monash University Malaysia, Selangor, Malaysia; andrian.liem@monash.edu; https://orcid.org/0000-0002-1746-7235.
2. Karmia A. Pakingan
   Department of Psychology, De La Salle University, Manila, Philippines; karmia.pakingan@dlsu.edu.ph; https://orcid.org/0000-0002-8830-7669.
3. Melissa R. Garabiles
   Department of Psychology, De La Salle University, Manila, Philippines; Scalabrini Migration Center, Quezon City, Philippines; melissargarabiles@gmail.com; https://orcid.org/0000-0002-2928-740X.
4. Hao Fong Sit
   Department of Psychology, The University of Hong Kong, Hong Kong (SAR), People's Republic of China; hfsit@connect.hku.hk; https://orcid.org/0000-0003-3076-1049.
5. Sebastian Burchert
   Division of Clinical-Psychological Intervention, Department of Education and Psychology, Freie Universität Berlin, Berlin, Germany; s.burchert@fu-berlin.de; https://orcid.org/0000-0003-3126-5485.
6. Agnes I.F. Lam
   Department of Communication & Centre for Macau Studies, University of Macau, Macau (SAR), China; agneslam@um.edu.mo; https://orcid.org/0000-0002-4492-8877.
7. Brian J. Hall*
   New York University (Shanghai), Shanghai, People's Republic of China; Johns Hopkins Bloomberg School of Public Health, USA; bjh9622@nyu.edu; https://orcid.org/0000-0001-9358-2377.

**corresponding author*

Supplements: 5

Liem A, Pakingan KA, Garabiles MR, Sit HF, Burchert S, Lam AIF and Hall BJ (2022) Evaluating the Implementation of a Mental Health App for Overseas Filipino Workers in Macao China: A Mixed-Methods Study of Stakeholders' Perspectives. Front. Psychiatry 13:836156. doi: 10.3389/fpsyt.2022.836156

Final

SUPPLEMENTS

Supplement 1

Online questionnaire for local NGO staff members and e-helpers in the Philippines

As part of the evaluation process, we would like to know your perspective on Kumusta Kabayan / Step by Step program. There will be several parts that cover different aspects and will take 10-15 minutes to complete this survey. This survey is anonymous, means your identity will not be recognized. Therefore, please give your honest responses to each item. Thank you in advance for your participation. By clicking “>>” you agree to anonymously participate in this evaluation.

Organizational Climate

Below are some statements related to the atmosphere of your organizational / institution / group. Please choose one response that represent your evaluation to each item. The response choices are: Strongly disagree (1), Somewhat disagree (2), Neither agree nor disagree (3), Somewhat agree (4), Strongly agree (5)

Part 1 (Mission) [only for team in Macao]

M1. The Kumusta Kabayan / Step by Step program fits in my organization / institution’s mission.

M2. The Kumusta Kabayan / Step by Step program fits in my organization / institution’s available services.

M3. The Kumusta Kabayan / Step by Step program fits with my organization / institution’s target users.

Part 2 (Teamwork)

The following statements are related to your general work environment, not just related to Kumusta Kabayan.

T1. Our team is willing to take a chance on a good idea.

T2. I can usually count on getting assistance from my supervisor when I have a difficult assignment.

T3. I can usually count on getting assistance from my colleagues when I have a difficult assignment.

T4. We are encouraged to speak our minds, even if it means disagreeing with our supervisor.

T5. We have regular meetings as a team to problem-solve about particular clients.

Part 3 (Infrastructure support)

S1. The Kumusta Kabayan / Step by Step program is compatible with currently available equipment and technology at my organization / institution.

S2. The Kumusta Kabayan / Step by Step program is compatible with currently available human resources availability and skills at my organization / institution.

S3. My organization / institution can financially support the Kumusta Kabayan / Step by Step program.

Final

Awareness and Concern

Below are statements about your awareness to Kumusta Kabayan / Step by Step program. Please choose one response that most reflect your feeling. The responses are:
Not at all true (1), Slightly true (2), Somewhat true (3), Very true (4)

Part 1 (Awareness)

Aw1. I do not know what a Kumusta Kabayan / Step by Step program is.

Aw2. I am aware that Kumusta Kabayan / Step by Step program addresses depressive symptoms.

Aw3. I can distinguish the formats between the two intervention groups (5-session and psychoeducation).

Part 2 (Concern)

C1. I do not believe doing Kumusta Kabayan / Step by Step program is so important.

C2. I do not care about the Kumusta Kabayan / Step by Step program.

C3. I am not sure why some peoples consider the Kumusta Kabayan / Step by Step program is important.

C4. I do not know why treating Filipino workers' depressive symptoms is so important for my organization / institution to address.

Part 3 (Interest)

I1. I am interested in learning more how to make the Kumusta Kabayan / Step by Step program available to more people.

I2. I would like to explore the possibility of improving the Kumusta Kabayan / Step by Step program in my organization / institution.

Perceived advantage and complexity

Below are some statements related to the advantage and complexity of the Kumusta Kabayan / Step by Step program. Please choose one response that represents your thought on the item. The possible responses are:
Strongly disagree (1), Somewhat disagree (2), Neither agree nor disagree (3), Somewhat agree (4), Strongly agree (5)

Part 1 (Relative advantage)

Ad1. The Kumusta Kabayan / Step by Step program would be more effective in treating depressive symptoms than currently available treatment for Filipino workers in Macao.

Ad2. The Kumusta Kabayan / Step by Step program can improve the mental health of Filipino workers in Macao.

Ad3. The Kumusta Kabayan / Step by Step program is better than currently available depression treatment in our organization for Filipino workers in Macao.

Final

Part 2a (Complexity – Organizational level)

Cp1. The Kumusta Kabayan / Step by Step program would be difficult for our organization / institution to explain to potential Filipino migrant worker users.

Part 2b (Complexity – Individual level)

Cp2. The Kumusta Kabayan / Step by Step program would be hard for Filipino migrant workers to understand.

Cp3. The Kumusta Kabayan / Step by Step program would be hard for Filipino workers to apply in their daily lives.

Level of Success

The goals of the Kumusta Kabayan / Step by Step program were to: 1) reduce depressive symptoms, 2) reduce anxiety symptoms, and 3) increase well-being of Filipino workers in Macao. Based on your experience and observation, which of the following statements best describes your impression of the program successfulness? (Please choose ONLY one statement, which 1 represents very unsuccessful and 8 represents very successful)

1. The Kumusta Kabayan / Step by Step program not only failed to meet its goals, but it caused a loss of resources and create other problems.
2. The Kumusta Kabayan / Step by Step program achieved none of the goals.
3. The Kumusta Kabayan / Step by Step program achieved one or two goals.
4. The Kumusta Kabayan / Step by Step program achieved none of its goals, but some Filipino migrant workers wish to continue its use.
5. Although the Kumusta Kabayan / Step by Step program achieved none of its goals, but it provided other benefits for Filipino migrant workers.
6. The Kumusta Kabayan / Step by Step program achieved one or two goals, but caused other problems.
7. The Kumusta Kabayan / Step by Step program achieved all of its goals.
8. The Kumusta Kabayan / Step by Step program fulfilled all of its goals and also provided other benefits for Filipino migrant workers and/or for other stakeholders.

General feedback and suggestion

If you have any feedback and suggestion related to Kumusta Kabayan / Step by Step program, please write them here.

Thank you for your participation. If you have any question, please send email to stepbystepmacao@gmail.

Final

Supplement 2

Interview guideline for app users (OFWs in Macao)

ENGLISH	FILIPINO
**INTRODUCTION**<br>I am ____, I am part of the Kumusta Kabayan – Caritas Macau program. I am calling you to interview you about the Kumusta Kabayan program. Your answers will be used to help us improve on the program. The interview will last 20-30 minutes. I will be recording the interview. After the interview, you will be given a 100 MOP worth of Jollibee voucher.<br>Your participation is voluntary, which means you will decide if you will join or not. If you decide not to join, there will be no negative effect on you. You may decide to stop participating in the interview or you may choose that we do not use your data at any time, even if the interview has already started. All the information you will give will be confidential. Your name will not appear on any output. Only the researchers will know the information that you will share, and we will not show the information to anyone. We will keep the information in password-protected computers.<br>Do you have questions or is there anything that is unclear? [wait for answer]<br>Do you agree to join the interview? It will only last 20-30 minutes. [If participant agrees, proceed with interview. If not, ask why then say thank you]	**INTRODUCTION**<br>Ako po si ____, kasama po ako sa Kumusta Kabayan – Caritas Macau program. Tumatawag po ako para ma-interview kayo tungkol sa Kumusta Kabayan program. Ang mga isasagot mo ay gagamitin para mapaganda namin ang programa. Tatagal po ang interview ng 20-30 minuto. Irerecord ko po ang interview. Pagkatapos po ng interview, ikaw ay makatatanggap ng 100 MOP na Jollibee voucher.<br>Boluntaryo ang pagsali mo sa interview, ibig sabihin, ikaw ang magpapasya kung sasali ka ba o hindi. Kung mag-desisyon kang hindi sasali, walang magiging negatibong epekto sa iyo. Pwede kang tumigil sa pagsali o piliing huwag ipagamit ang iyong data sa kahit na anong oras kahit na nag-umpisa na ito. Ang lahat ng impormasyong ibibigay mo ay magiging confidential. Hindi lalabas ang iyong pangalan sa mga output. Ang mga researchers lang makakaalam ng mga impormasyon, at hindi namin ipapakita ito sa kahit na sino. Itatago namin ang mga impormasyon sa mga password-protected na computers.<br>May tanong po ba kayo o may hindi po malinaw? [wait for answer] Pumapayag po ba kayong sumali sa interview? Tatagal lang po ito ng 20-30 minuto [If participant agrees, proceed with interview. If not, ask why and say thank you]
**WARMING UP / OPENING**<br>1. When did you install / use Kumusta Kabayan for the first time? [it's okay if not remember the detail, such as "early Aug"]<br>2. Did you install the app directly on your phone or via the mobile website version?<br>**3.** What was on the screen? OR How did the screen look like? [to check whether s/he was in the 5-session intervention or psychoeducation group]	**WARMING UP / OPENING**<br>1. Kailan mo unang ininstall o ginamit ang Kumusta Kabayan? [it's okay if not remember the detail, such as "early Aug"]<br>2. Ininstall niyo po ba ang app sa phone niyo o ginamit niyo ang mobile website version?<br>**3.** Ano yung nakalagay sa screen? OR Anong itsura ng screen? [to check whether s/he was in the 5-session intervention or psychoeducation group]

Final

ENGLISH	FILIPINO
**MAIN QUESTIONS**	**MAIN QUESTIONS**
1. What did you think about Kumusta Kabayan before installing / using it?	1. Bago mo ininstall o ginamit ang Kumusta Kabayan, anong naisip mo tungkol sa app?
2. After you installed / used it, did the program match with your idea? (please elaborate)	2. Matapos mong iinstall o gamitin ito, nag-match ba yung naisip mo tungkol dito at ang karanasan mo sa paggamit nito?
3. When did usually you use the app (eg during the day, evening; weekday, weekend; etc)? For how long?	3. Kailan mo madalas gamitin ang app (e.g., sa araw, gabi; weekday, weekend; etc)? Gaano katagal?
4. What did language version you use? Was it easy or difficult to understand?	4. Anong language version ang ginamit mo? Madali ba o mahirap itong intindihin?
5. What did you like from the app (ask them to elaborate their answer)?	5. Anong nagustuhan mo sa app (ask to elaborate)?
a. What did you learn from Kumusta Kabayan? (eg new skill, technique, knowledge, networks/supports?)<br>***Ask 5b, 5c, and 5d only to those who were part of 5-session group:**	a. Anong natutunan mo sa Kumusta Kabayan? (e.g., new skill, technique, knowledge, networks/supports)<br>***Tanungin lang ang 5b, 5c, at 5d sa mga nasa 5-session group:**
b. Probe if not mentioned: Did you like<br>i. the stories? [if yes: what did you like about the stories?]<br>ii. The characters? [if yes: what did you like about the characters?]<br>iii. The activities? [if yes: what did you like about the activities?]	b. Probe if not mentioned: Nagustuhan mo ba:<br>i. Ang mga istorya? [if yes: anong nagustuhan mo sa mga istorya?]<br>ii. Ang mga characters? [if yes: anong nagustuhan mo sa mga characters?]<br>iii. Ang mga activities? [if yes: anong nagustuhan mo sa mga activities?]
c. Did you use the features (ie Mood Tracker) in Kumusta Kabayan? [If not ask: What were some reasons they did not use these features?]	c. Ginamit mo ba ang mga features (e.g., Mood Tracker) sa Kumusta Kabayan? [Kung hindi, anong mga dahilan kung bakit mo hindi ginamit ang mga ito?]
d. Did you practice the suggested activities like breathing exercise, grounding exercise, or doing something enjoyable? [If not, ask: what were some reasons not to practice these activities? If yes, ask: how was it?]	d. Nag-practice ka ba ng mga activities, tulad ng breathing exercise, grounding exercise, o paggawa ng isang bagay na nakaka-enjoy? [Kung hindi: anu-anong mga dahilan at hindi ka nagpractice? Kung oo: kumusta ito?]
6. Did you think that using Kumusta Kabayan helped you in anyway? [wait for answer] If yes, how did it help you?	6. Sa tingin mo ba, nakatulong ang Kumusta Kabayan sa iyo sa kahit na anong paraan? [wait for answer] Kung oo, paano ito nakatulong sa iyo?
7. Did you encounter any problems when using Kumusta Kabayan? If yes, could you please elaborate?	7. Nagka-problema ka ba sa paggamit ng Kumusta Kabayan? Kung oo: anu-ano ito?
8. Did you receive a call from e-helper? Did you	8. Nakatanggap ka ba ng tawag mula sa isang e-

Final

ENGLISH	FILIPINO
pick it up? [If not, ask: what were some reasons not to pick the call? If yes, what do you think of the call or message from the e-helper?	helper? Sinagot mo ba ang tawag? [Kung hindi: anong dahilan at hindi mo tinanggap ang tawag? Kung oo: anong masasabi mo sa tawag o message na nakuha mo sa e-helper?]
9. ***Ask only to those who were part of the 5-session group:** When was the last time you use / open the app? (compared with #5 to check the consistency)* [if the user discontinued to use the app] Could you tell us some of the reasons you might have that hinder or make you difficult to continue the use of Kumusta Kabayan?*	9. ***Tanungin lang sa mga nasa 5-session group:** Kailan mo huling ginamit o binuksan ang app? [kung hindi na ginagamit ang app: Anong mga dahilan mo sa pagtigil sa paggamit ng app?]
10. We really want to improve Kumusta Kabayan so that OFWs will be interested and motivated to use it. In your opinion, what are the changes that need to be done in the app?	10. Gusto namin na pagandahin ang Kumusta Kabayan para ang ibang mga OFW ay maging interesado at ma-engganyong gamitin ito. Sa iyong opinion, anu-ano ang mga pagbabago sa app na kailangang gawin?
11. In the future, would you recommend Kumusta Kabayan to your OFW fellows? Why?	11. Irerekomenda mo ba ang Kumusta Kabayan sa iyong kapwa OFW? Bakit?
12. Last question, do you have any comment or suggestion about Kumusta Kabayan in general?	12. Huling tanong na, may masasabi ka pa ba o mga suhestiyon tungkol sa Kumusta Kabayan?
**CLOSING**<br>Thank you very much! Your answers will help us a lot. You may claim your 100 MOP Jollibee voucher at the Philippine Consulate. Please go there on Sunday from 10am-5pm.	**CLOSING**<br>Maraming salamat po! Malaking tulong po ang mga sagot ninyo. Pwede niyo pong makuha ang 100 MOP Jollibee voucher niyo sa Philippine Consulate. Pumunta po kayo sa Linggo, mula 10am-5pm.

* = Only for users in the 5-session group

Final

Supplement 3
Characteristics of interviewees

Order	Interview date	#	Group	Status	Sex	Age	Marital status	Educational background	Total months as OFW	Total months as OFW in Macao	Housing	Type of job	Monthly salary (MOP)	Working hours/week	Insurance	Interview duration (in minutes)
1	19/10/20	43	PE	U	M	32	1	6	11	5	Live-out	6	7,500	48	4	15
2	22/10/20	44	PE	C	F	42	2	3	240	120	Live-in	14	4,300	96	2	15
3	27/10/20	41	PE	U	F	43	2	3	7	7	Live-in	14	3,000	78	1	18
4	29/10/20	40	PE	U	F	50	2	6	324	120	Live-in	14	5,200	60	3	37
5	1/11/20	28	5S	C	F	49	2	6	84	57	Live-out	14	4,300	72	3	20
6	11/11/20	6	5S	U	F	42	1	2	84	60	Live-out	14	4,000	72	1	24
7	15/11/20	168	5S	C	F	36	1	2	60	60	Live-out	14	4,200	72	3	20
8	17/11/20	50	PE	U	F	38	2	2	36	12	Live-in	14	4,200	72	3	31
9	17/11/20	53	5S	U	F	42	2	2	72	43	Live-in	14	4,300	48	3	41
10	21/11/20	94	PE	C	M	26	1	6	12	12	Live-out	6	6,000	72	2	30
11	22/11/20	139	PE	C	F	28	4	3	72	60	Live-out	14	4,000	54	3	15
12	30/11/20	153	5S	U	M	41	4	6	180	180	Live-out	7	6,500	44	1	30
13	8/12/20	197	PE	U	F	38	2	2	19	19	Live-in	14	3,500	48	3	15
14	8/12/20	194	5S	C	F	32	4	2	35	15	Live-out	14	3,200	78	3	35
15	15/12/20	170	PE	U	M	25	1	6	17	11	Live-out	4	6,600	96	2	35
16	17/1/21	207	5S	U	F	50	6	2	48	48	Live-out	14	4,000	78	1	31
17	24/1/21	283	PE	U	M	42	2	6	29	29	Live-out	2	6,500	54	5	20
18	24/1/21	289	5S	C	F	43	2	5	16	16	Live-in	14	4,200	90	3	30

Order	Interview date	#	Group	Status	Sex	Age	Marital status	Educational background	Total months as OFW	Total months as OFW in Macao	Housing	Type of job	Monthly salary (MOP)	Working hours/week	Insurance	Interview duration (in minutes)
19	25/1/21	128	PE	C	F	23	1	6	13	13	Live-out	13	6,000	48	2	20
20	25/1/21	232	5S	U	F	38	1	5	24	24	Live-in	14	4,500	60	4	30
21	26/1/21	245	PE	U	F	49	4	2	20	11	Live-out	14	5,000	60	3	10
22	29/1/21	159	5S	C	F	34	2	2	36	23	Live-in	14	4,000	90	1	31
23	30/1/21	198	PE	U	F	37	2	2	7	2	Live-in	14	3,800	48	1	20
24	30/1/21	247	5S	C	F	46	2	2	10	10	Live-in	14	3,800	72	3	20
25	9/2/21	265	PE	C	F	45	6	2	24	24	Live-in	14	4,000	48	1	20
	M					39			59	39			4,664	66		25
	SD					8			78	43			1,182	16		8

Notes. Group: 5S = 5-session, PE = Psychoeducation. Status: C = Completed, U = Uncompleted. Sex: F = Female, M = Male. Marital status: 1 = Single-never married, 2 = Married, 3 = Partnered but not married, 4 = Separated, 5 = Legally separated, 6 = Widowed. Educational Background: 1= Elementary, 2 = High School, 3 = Technical/Vocational, 4 = 2-year Associate Degree", 5 = Some College, 6 = Graduated College (Bachelor's Degree), 7 = Master's Degree or higher. Housing: Live-in = Stay with the employer, Live-out = Not stay with employer. Type of job: 2 = Manufacturing, 4 = Construction, 6 = Hotels, restaurants and similar activities, 7 = Transport, storage and communication, 13 = Recreational, cultural, gaming & other services, 14 = Domestic work. MOP 1 ≈ USD 0.124 (exchange rate in July 2021). MOP = Macao Pataca. Working hours/week for 6 days/week ≈ 11 hours/day. Insurance: 1 = I do not know, 2 = No insurance, 3 = Medical insurance (standard – for accidents during work), 4 = Medical insurance (comprehensive – general health), 5 = No insurance but the employer reimburses the expenses when I get sick.

Final

Supplement 4

Characteristics of non-interviewees (*N* = 161)

**Variables**	***f***	***M (SD)***	***Med***
**Group**			
5-session	76		
Psychoeducation	85		
**Sex**			
Female	135		
Male	26		
**Age**		39 (9)	38
**Marital status**			
Single-never married	47		
Married	81		
Partnered but not married	9		
Separated	17		
Legally separated	1		
Widowed	4		
Did not answer	2		
**Educational background**			
Elementary	1		
High School	33		
Technical/Vocational	14		
2-year Associate Degree	14		
Some College	16		
Graduated College (Bachelor's Degree)	35		
Did not answer	48		
**Total months as OFW**		53 (59)	36
**Total months as OFW in Macao**		38 (35)	25
**Housing**			
Live-in	35		
Live-out	124		
Did not answer	2		
**Type of job**			
Agriculture, farming of animals, hunting and forestry	1		
Manufacturing	5		
Hotels, restaurants and similar activities	28		
Education	2		
Health and social welfare	1		

Final

Variables	*f*	*M (SD)*	*Med*
Recreational, cultural, gaming & other services	3		
Domestic work	69		
Did not answer	52		
**Monthly salary (MOP)**		5,072 (3,026)	4,000
**Working hours/week**		61 (17)	60
**Insurance**			
I do not know	59		
No insurance	35		
Medical insurance (standard – for accidents during work)	31		
Medical insurance (comprehensive – general health)	27		
No insurance but the employer reimburses the expenses when I get sick	8		
Did not answer	1		

Notes. Housing: Live-in = Stay with the employer, Live-out = Not stay with employer. *f* = Frequency. *M* = Mean. *SD* = Standard Deviation. *Med* = Median.

Final

Supplement 5

Promotional strategy samples

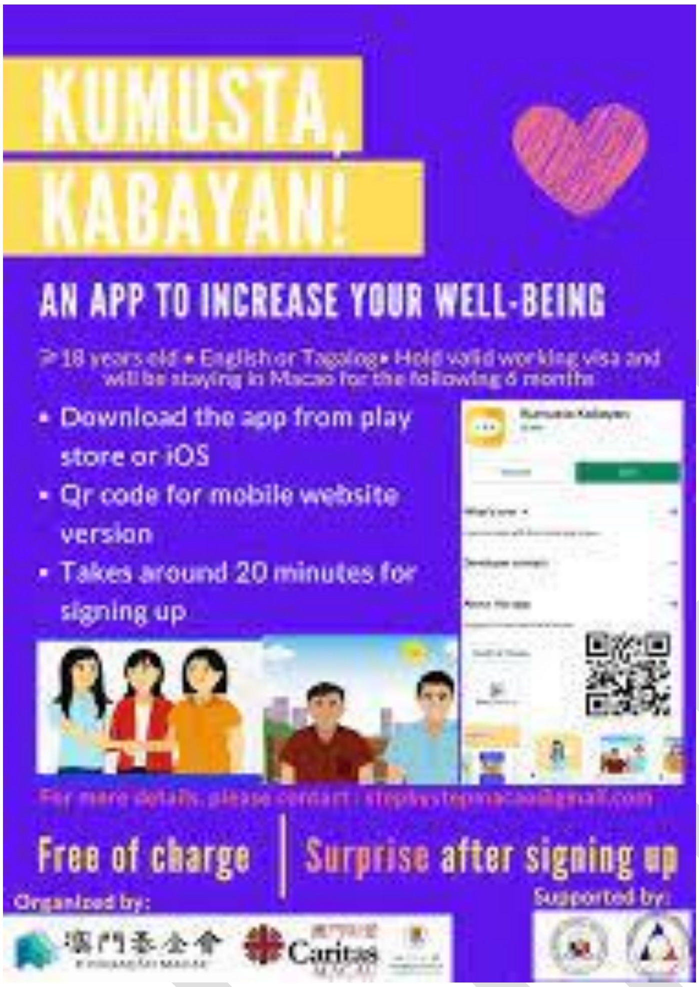

A. Poster to promote the program

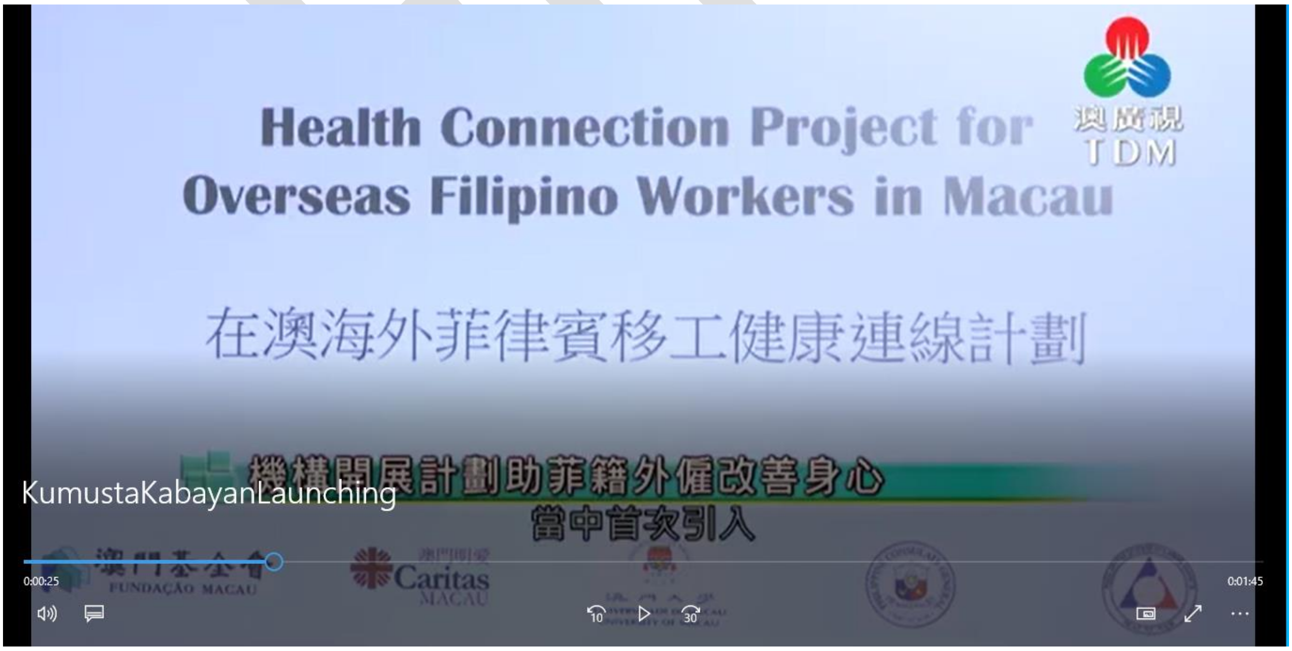

B. Launching of program that was covered by the local news channel
